# Production of FAME biodiesel in *E. coli* by direct methylation with an insect enzyme

**DOI:** 10.1038/srep24239

**Published:** 2016-04-07

**Authors:** Saken Sherkhanov, Tyler P. Korman, Steven G Clarke, James U. Bowie

**Affiliations:** 1 Department of Chemistry and Biochemistry UCLA-DOE Institute Molecular Biology Institute, University of California, Los Angeles, USA.

## Abstract

Most biodiesel currently in use consists of fatty acid methyl esters (FAMEs) produced by transesterification of plant oils with methanol. To reduce competition with food supplies, it would be desirable to directly produce biodiesel in microorganisms. To date, the most effective pathway for the production of biodiesel in bacteria yields fatty acid ethyl esters (FAEEs) at up to ~1.5 g/L. A much simpler route to biodiesel produces FAMEs by direct *S*-adenosyl-*L*-methionine (SAM) dependent methylation of free fatty acids, but FAME production by this route has been limited to only ~16 mg/L. Here we employ an alternative, broad spectrum methyltransferase, *Drosophila melanogaster* Juvenile Hormone Acid *O*-Methyltransferase (*Dm*JHAMT). By introducing DmJHAMT in *E. coli* engineered to produce medium chain fatty acids and overproduce SAM, we obtain medium chain FAMEs at titers of 0.56 g/L, a 35-fold increase over titers previously achieved. Although considerable improvements will be needed for viable bacterial production of FAMEs and FAEEs for biofuels, it may be easier to optimize and transport the FAME production pathway to other microorganisms because it involves fewer enzymes.

Increasing energy consumption and the detrimental environmental impact of fossil fuels has led to increased interest in developing sustainable and renewable sources of energy. The utilization of engineered microorganisms to produce chemicals from renewable biomass is a promising alternative to petroleum-derived fuels and chemicals. Fatty acid derived compounds are particularly promising because fatty acid derivatives are highly reduced, aliphatic compounds with high energy density that are not miscible with water^1^. Notably, their similarity to diesel fuels makes them compatible with existing infrastructure. As a result, many strategies have been developed to overproduce microbial fatty acids and then further convert the fatty acids into biofuels such as alkanes, fatty alcohols, and fatty acid methyl or ethyl esters^2^,^3^,^4^,^5^,^6^,^7^,^8^.

Microbial production of fatty acid methyl or ethyl esters (FAME, FAEE respectively) is of particular interest because FAME and FAEE are the main component of biodiesel currently in use. Typically, biodiesel is made by transesterification of triacylglyceride oils extracted from renewable biomass with short chain alcohols (e.g methanol or ethanol) using an alkaline catalyst^9^. However, the use of feedstock oils needed for biodiesel production is a major obstacle for the broader use of biodiesel due to lack of arable land and competition with the food supply. Therefore, a possible alternative to plant and animal oil-based biodiesel is the direct biosynthetic production of biodiesel in metabolically engineered microorganisms (reviewed in^10^).

Steinbuchel and co-workers were the first to develop a pathway for the production of FAEE biodiesel in *E. coli,* and their approach was further developed by the Keasling group for increased yields of FAEE and fatty alcohols^2^,^4^,^7^. To produce FAEEs, two orthogonal pathways were introduced that simultaneously generated ethanol and fatty acyl-CoA. In the last step, ethanol and fatty acyl-CoA were then condensed to the FAEE using a wax ester synthase (Fig. 1)^2^,^7^. After optimization, titers as high as 1.5 g of long chain FAEEs per liter of culture were obtained.

As a more straightforward approach to produce biodiesel in microorganisms, the Lykidis group attempted to produce FAMEs in *E. coli* through direct methylation of fatty acids by the action of an *S-*adenosyl-*L-*methionine (SAM) dependent bacterial methyltransferase from *M. marinum*^3^. The Lykidis pathway has the advantage of being much simpler than the FAEE production pathway by using endogenous compounds (SAM and fatty acids) produced in *E. coli.* Nevertheless, the FAME titers obtained were nearly two orders of magnitude lower than the FAEE titers (16 mg/L). The low level of FAME production is likely due to the high specificity of the methyltransferase employed, which prefers rare fatty acids containing a 3-hydroxy group^3^.

We hypothesized that if we could find a broad range fatty acid methyl transferase, perhaps we could improve upon the Lykidis approach for FAME production. Here we show that *Drosophila melanogaster* Juvenile Hormone Acid *O*-Methyltransferase (*Dm*JHAMT) has broad specificity for medium chain free fatty acids and can be used to produce FAMEs in *E. coli*. By introducing *Dm*JHAMT to engineered *E. coli* strains tolerant to high levels of endogenously produced medium chain fatty acids, we observed *in vivo* FAME production^11^. Enriching the endogenous SAM pool further increased FAME production with final titers showing a 35-fold increase from titers previously reported^3^.

## Results and Discussion

### *Dm*JHAMT is robustly expressed and broadly active on medium chain fatty acids

Several SAM-dependent juvenile hormone acid methyltransferases have been previously found to methylate insect sesquiterpenoid hormones that play central roles in the development and growth of these organisms^12^,^13^,^14^*. D. melanogaster* Juvenile hormone acid *O-*methyltransferases (*Dm*JHAMT) appeared to be a promising enzyme for FAME production because it showed some activity with unbranched saturated medium and long-chain fatty acids such as lauric and palmitic acids, and could be expressed in *E. coli*^13^.

**Figure 2:**
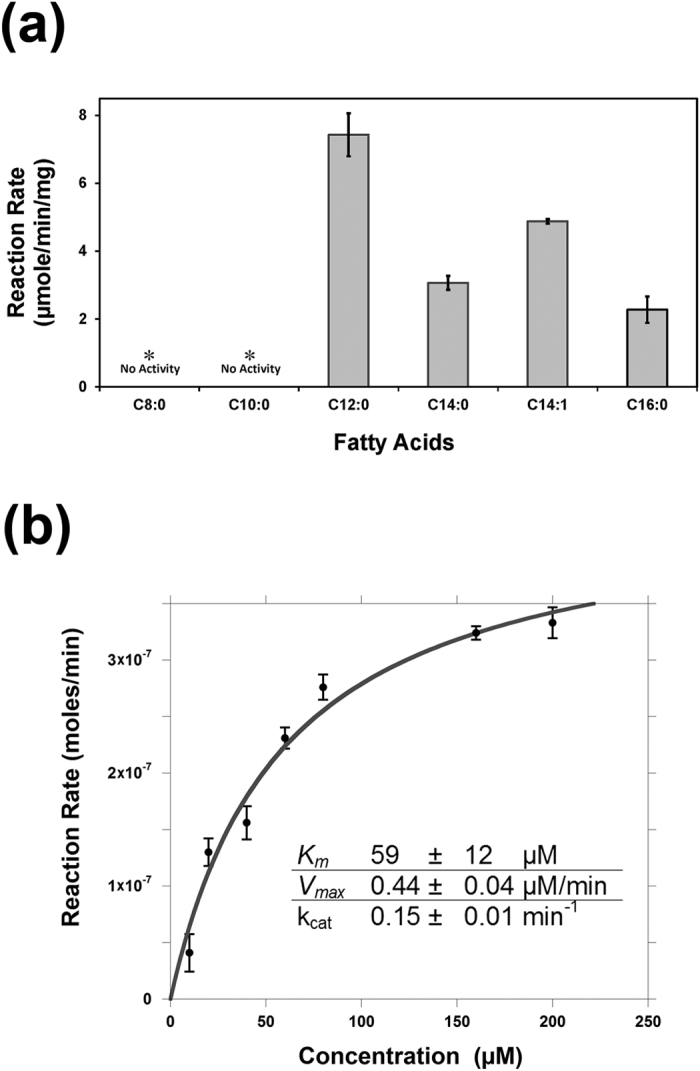
*Dm*JHAMT methylates a broad spectrum of fatty acids. (**a**) *Dm*JHAMT methylates straight and branched medium-chain fatty acids *in vitro*. The reaction rates observed using 40 μM of each fatty acid are shown. (**b**) Kinetic analysis of recombinant *Dm*JHAMT activity with lauric acid.

We expressed the *Dm*JHAMT protein recombinantly in *E. coli* to investigate its substrate specificity. *Dm*JHAMT expression was robust in *E. coli* (up to 200 mg of protein per liter of culture) with no apparent effect on cell growth. As shown in Fig. 2a, *Dm*JHAMT is active on fatty acids ranging in size from C12 to C16. We saw no activity with shorter chain, C8:0 and C10:0, fatty acids, however. *Dm*JHAMT is most active on medium chain fatty acids, showing the highest activity with lauric acid (C12:0) among the substrates tested. The kinetic parameters with lauric acid were *K*_*m*_ = 59 μM and k_cat_ = 0.15 min^−1^ (Fig. 2b). Although the low k_cat_ indicates that the enzyme is not very efficient with these non-natural substrates, the high expression and broad specificity of *Dm*JHAMT suggested that it might be effective at producing FAMEs, particularly medium chain FAMEs, in *E. coli*.

### *Dm*JHAMT produces FAME biodiesel in *E. coli*

*E. coli* has been utilized as a host for over-production of free fatty acids (FFAs) of various lengths and properties^2^,^5^,^15^,^16^,^17^,^18^,^19^,^20^,^21^,^22^,^23^,^24^. Introduction of bacterial and plant acyl-ACP thioesterases in a Δ*fadD* mutant *E. coli* strain defective in fatty acid degradation allows overproduction of free fatty acids by liberating fatty acids attached to acyl-carrying proteins (ACPs), while simultaneously removing acyl-ACP mediated regulation of the fatty acid biosynthesis pathway, effectively redirecting lipid biosynthesis into free fatty acid production^25^. Since *Dm*JHAMT is most active with medium chain FFAs, we opted to utilize the acyl-ACP thioesterase from *Umbellularia californica* (BTE), which has a preference for medium chain fatty acids and leads to accumulation of lauric acid when expressed in an *E. coli ∆fadD* strain^5^,^26^.

**Figure 3:**
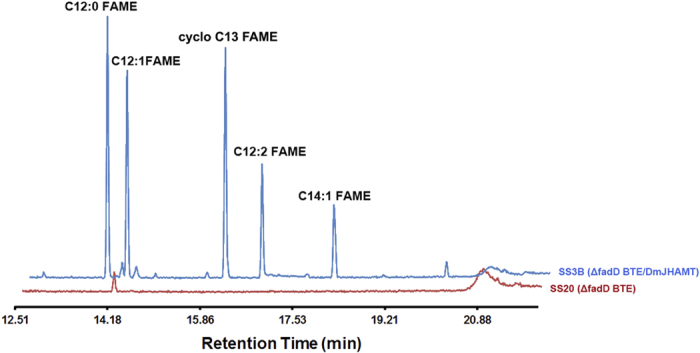
Production of FAMEs in *E. coli.* GC-MS analysis of FAMEs produced in strain SS3B (*∆fadD* BTE/*Dm*JHAMT; blue), compared to the control strain, SS20 (*∆fadD* BTE; red) that does not produce *Dm*JHAMT. Most of the FAMEs produced in SS3B contain 12-carbon acyl groups with majority being methyl laurate. C12:0, methyl laurate; C12:1, cis-5-dodecenoic acid methyl ester; cyclo C13, cyclopropanedodecanoic acid methyl ester; C12:2, 3,6-dodecadienoic acid methyl ester; C14:1, methyl myristoleate.

We first prepared strain SS3B (*∆fadD Dm*JHAMT/BTE) bearing a *∆fadD* mutation and expressing *Dm*JHAMT and BTE from plasmids (Table 1). In strain SS3B we observed relatively high production of medium-chain fatty acid methyl esters (Fig. 3). The initial titer of FAMEs was 240 ± 15 mg/L of culture, already a dramatic improvement over prior results^3^. Since medium-chain FAMEs are somewhat volatile, we added dodecane as an organic overlay at the stationary phase to trap the FAMEs, which further increased the titer of FAMEs to 312 mg/L of culture^2^. The majority of FAMEs contained 12-carbon acyl chains (73%), mostly unsaturated C12 methyl laurate (Fig. 3).

While a high level of FAMEs were produced, we were surprised to find that strain SS3B (*∆fadD Dm*JHAMT/BTE) still produced a considerable amount of free fatty acids (FFA) that were not methylated (860 ± 20 mg/L of culture). Indeed a majority of the FFAs generated in strain SS3B were not converted into FAMEs. We therefore sought to increase the conversion of the excess FFAs to FAMEs.

### Increasing SAM levels

**Figure 4:**
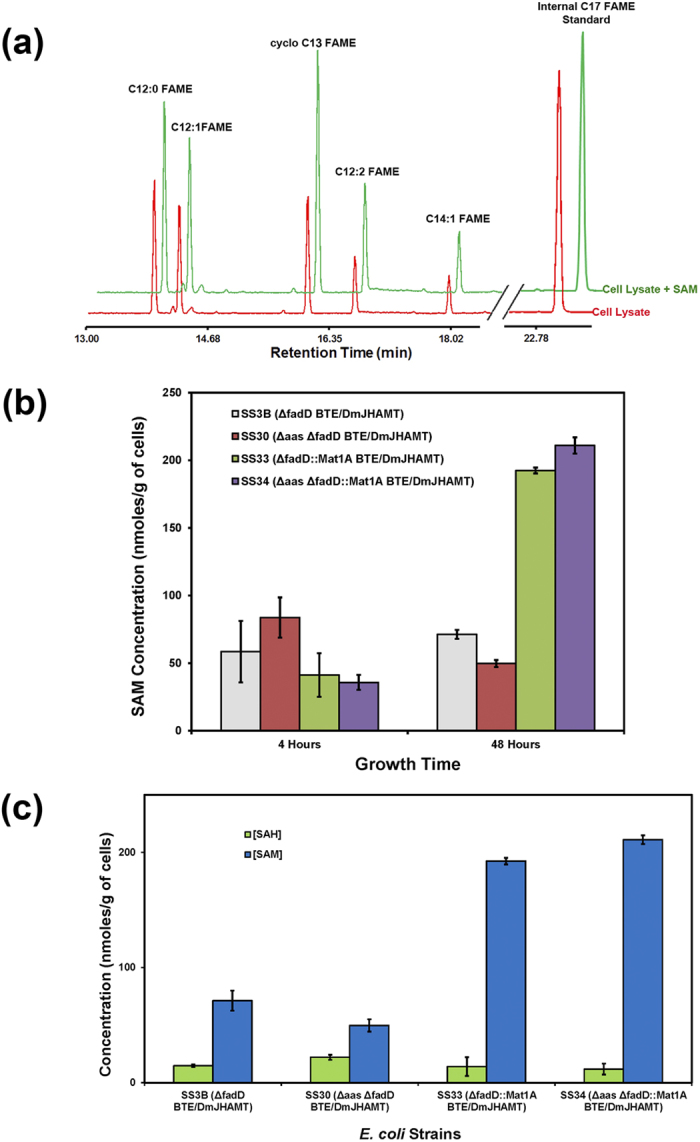
SAM is a limiting factor for *in vivo* production of FAMEs. (**a**) FAME-producing strain, SS3B (*∆*fadD BTE/*Dm*JHAMT; red), was lysed and exogenous 500 μM SAM was added to the lysate and incubated for 40 min at 25 °C (green). An internal standard (methyl heptadecanoate) was added to cell culture prior to lysis. (**b**) SAM levels in strains producing FAMEs. (**c**) SAM and SAH concentrations in the FAME-producing cell lines after 48 hours of growth. While SAH levels are similar in all strains, the concentration of SAM increases dramatically in Mat1A-carrying strains.

We hypothesized that SAM levels may be a limiting factor in the conversion of FFAs to FAMEs. To test the possibility that low SAM levels during stationary phase contributed to low FAME production, we lysed strain SS3B after two days of growth and supplemented the lysate with exogenous SAM. We observed increases in all FAME species indicating that the *Dm*JHAMT remained active but the SAM levels may be limiting (Fig. 4a).

To increase SAM production, we introduced the methionine synthase protein from rat liver, Mat1A, into *E. coli* strain SS3B^27^,^28^. Mat1A was shown to dramatically increase the intracellular.

SAM pool in *E. coli* cells^28^. Mat1A expression from a plasmid in strain SS4B (*∆fadD Dm*JHAMT/BTE/Mat1A) increased SAM levels 8.5-fold (from 73.3 to 636.8 nmoles per gram of cells) compared to the control strain SS3B (*∆fadD Dm*JHAMT/BTE) after two days of growth. Nevertheless, we found that Mat1A overexpression actually decreased both FFA and FAME titers. Mat1A overexpression may have unexpected deleterious effect on FAME production such as toxicity, competition for expression with other proteins, high metabolic ATP demand for SAM production, or the complication of harboring three different plasmids, among other possibilities^29^.

To simplify the system and reduce the expression of Mat1A, we incorporated a single copy of the Mat1A gene into the *E. coli* genome under the control of a T7 promoter. When Mat1A was incorporated into the genome, we observed ~3-fold increase of SAM levels in strain SS33 (*∆*fadD::Mat1A BTE/*Dm*JHAMT) compared to SS3B (*∆*fadD BTE/*Dm*JHAMT) after two days of growth (192 ± 3 nmoles SAM per gram of cells compared to 71 ± 19 nmoles per gram of cells in control the strain, Fig. 4b). More importantly, we saw a 19% increase in FAME production, from 312 mg/L to 370 mg/L in cells carrying Mat1A in the genome. In addition, this strain had a higher ratio of SAM to *S-*adenosylhomocysteine (SAH), a by-product SAM-dependent methylation and a potent inhibitor of methyltransferases (Fig. 4c). While the levels of SAH were similar in these strains, the levels of SAM showed considerable increases in Mat1A-carrying strains after 48 hours of growth^30^. Overall, Mat1A expression improved the production of FAMEs.

### Δ*aas* further increases the FAMEs titers in *E. coli*

**Figure 5:**
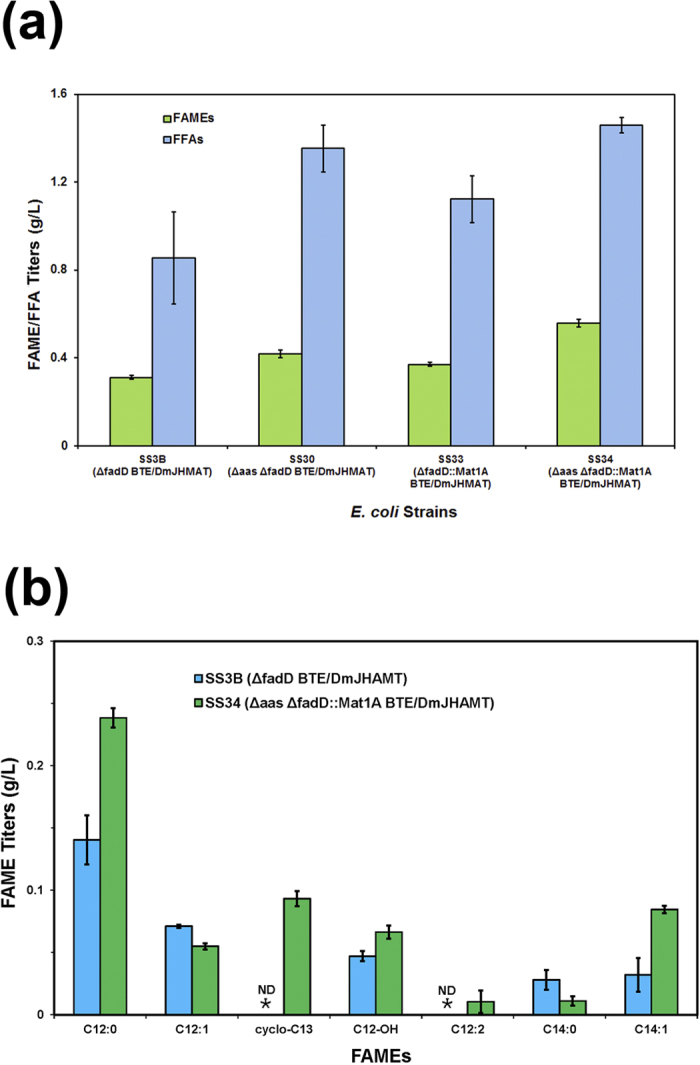
Δ*aas* and Mat1A increase FAME production in medium chain FA-producing *E. coli* cells. (**a**) Total FAME and FFA titers in engineered *E. coli* strains overlayed with organic layer. (**b**) The distribution of different FAMEs produced in SS3B (ΔfadD BTE/*Dm*JHAMT) and SS34 (Δaas ΔfadD::Mat1A BTE/*Dm*JHAMT) strains. ND, not detected.

Short and medium chain FFAs are toxic to *E. coli* cells, most likely due to membrane stress^23^,^31^,^32^,^33^. It is possible that the production of excess FFAs in our strains is deleterious to FAME production. We recently reported that the deletion of the *aas* gene can alleviate medium chain FFA toxicity^11^. The Aas protein acts in a FFA salvage pathway that can incorporate exogenous medium chain FFAs directly into the lipid bilayer with deleterious consequences. We therefore attempted to reduce the toxicity of the medium chain fatty acids by deleting the *aas* gene in strain SS3B to produce strain SS34 (Δaas ΔfadD::Mat1A BTE/*Dm*JHAMT). Indeed strain SS34 showed an almost 50% increase in the FAME production (559 mg/L of culture) compared to the same strain with a wild type *aas* gene SS33 (ΔfadD::Mat1A BTE/*Dm*JHAMT). Overall, strain SS34 (Δaas ΔfadD::Mat1A BTE/*Dm*JHAMT) overlaid with a dodecane layer showed a 137% increased FAME titer from the starting strain SS3B (ΔfadD BTE/*Dm*JHAMT) (Fig. 5a,b).

### Spectrum of FAMEs produced

The best FAME producing stain, SS34, generated a broad spectrum of medium chain FAMEs. While the saturated aliphatic FAME methyl laurate (C12:0) was the most abundant, we also observed 3-hydroxy C12 (C12-OH), cyclopropanedodecanoic (cyclo-C13) acid, unsaturated straight chain (C12:1, C12:2 and C14:1) and saturated C14 (C14:0) fatty acid methyl esters (Fig. 5B). While saturated (C12:0, C14:0), unsaturated (C12:1, C14:1) and hydroxylated C12 fatty acids have been previously observed in BTE-expressing *E. coli* strains, the cyclopropanedodecanoic (cyclo-C13) and unsaturated C12:2 fatty acids are unusual products of bacterial fatty acid biosynthesis^5^,^11^,^26^. Bacterial phospholipid acyl chains are regularly modified as a response to temperature and increasing organic solvent concentrations and these fatty acids may be a by-product of phospholipase turn-over activity on membrane-disruptive 2-acyl glycerophosphoethanolamine (2-acyl-GPE), especially in Δ*aas* Δ*fadD* strains that lack both 2-acyl-GPE acyltransferase and fatty acid degradation pathways^34^,^35^,^36^. The broad specificity of *Dm*JHAMT and availability of SAM in the SS34 strain allows the conversion of these fatty acids to their methyl ester derivatives.

## Conclusion

We have engineered a strain of *E. coli* that produces FAMEs at levels comparable to the best FAEE production strain and at levels that are more than an order of magnitude greater than FAME titers previously attained^2^,^3^. Essential developments were the identification of a FFA methyltransferase that has broad specificity for fatty acids and could be overproduced in *E. coli* and deletion of the *aas* gene to reduce incorporation of toxic medium chain-length FFAs into the bilayer. The fact that more than half of the FFAs generated (1.45 g of FFAs vs 0.559 g FAME) are not methylated in the highest producing strain (SS34) suggests that there is still considerable room for improvement. We do not know why FFAs are not fully converted to FAMEs, but presumably some portion of the FFAs is sequestered from *Dm*JHAMT (e.g in the membrane) because there is still sufficient SAM (211 nmoles per g of cells) and active enzyme present after several days, yet FFAs remain. It is also possible that the FFAs that escape from the cell are not reabsorbed efficiently due to the Δ*fadD* mutation, the normal route for uptake of long-chain free fatty acids. Poor re-uptake may be particularly problematic for medium chain FFAs even with *fadD* intact^37^, so perhaps better results will be obtained with strains that can produce longer chain (C16 and C18) FFAs on which *Dm*JHAMT is active. Screening of other methyltransferases or the engineering of methyltranferases for broader specificity should allow for still further improvements and diversification of the FAME products. While heat of combustion and cetane number, a measure of diesel ignition quality, are similar in these molecules, increasing the proportion of unsaturated acyl groups in this biofuel mix adds beneficial properties such as lower cloud point and lower freezing temperature^38^,^39^. Current studies are underway to increase branched and unsaturated fatty acid yields in *E. coli* that could potentially be used in our one-step biodiesel production method^40^,^41^,^42^.

## Materials and Methods

### Materials

T4 DNA ligase and restriction endonucleases were obtained from New England Biolabs. DNA Polymerase Mastermix was from Denville Scientific. Ni-NTA Superflow, QIAprep Miniprep kits and QIAquick gel extraction kits were purchased from Qiagen. The λDE3 Lysogenization Kit was from EMD Chemicals. All reagents were from Sigma Aldrich except for LB agar and Terrific Broth which were obtained from Fisher Scientific. Oligonucleotide primers were synthesized by Valuegene and IDT. Gene sequencing and gene synthesis were performed by Genewiz. Assembly master mix (AMM) used for cloning was prepared as outlined in^43^. All DNA and protein concentrations were measured with Thermo Fisher Scientific Nanodrop 1000 Spectrophotometer. Colorimetric enzyme-coupled assays were performed in 96-well plates and measured with Molecular Devices SpectraMax M5 microplate reader.

### Plasmid construction

An 897-bp portion of *U. californica BTE (BTE)* gene lacking the thylakoid targeting sequence was prepared synthetically by Genewiz and the synthetic BTE gene was amplified by polymerase chain reaction (PCR) using primers XhoI-pBAD/p15A-BTE and NciI-pBAD/p15A-BTE (See Table I for primer sequences). The PCR product was digested with XhoI and NciI and ligated into XhoI/PstI digested plasmid pBAD/HisA/p15A^44^ to produce BTE-pBAD/p15A. A plasmid containing the mature *Dm*JHAMT gene was obtained from the Drosophila Genomics Resource Center at Indiana University. The *Dm*JHAMT gene was amplified (primers *Dm*JHAMT NdeI Forward and *Dm*JHAMT XhoI End) and cloned into pET-28a(+) (Novagen). The resulting plasmid was then digested with NcoI and XhoI to excise the *Dm*JHAMT gene and cloned into pET-15b (Novagen) to swap the His-tag to the C-terminus. The 5′-methylthioadenosine/S-adenosylhomocysteinenucleosidase (MTAN) gene from *E. coli* was amplified (primers 5′ *E. coli* SAH NdeI, 3′ *E. coli* SAH SacI) and cloned into NdeI/SacI digested pET-28a(+). The S-ribosylhomocysteinase (LuxS) gene from *Bacillus subtilis* was amplified (primers 5′ B. sub LuxS NheI and 3′ B. sub LuxS EagI) and cloned into NheI/EagI digested pET-28a(+). The resulting plasmid was used to clone the LuxS gene into pET-22b(+) with NdeI/Bpu1102I digestion and ligation. Rat liver S-adenosyl-L-methionine synthetase (Mat1A) was amplified with 5′ KpnI Mat1A Forward and 3′ XhoI Mat1A End primers from rat liver cDNA and cloned into pCDF-1b plasmid using XhoI and KpnI restriction sties.

To knock-in genes into the *E. coli* genome, we generated a plasmid, called pCDF-Cat, that contains a chloramphenicol resistance gene (*cat*) flanked by the FLP recognition target (FRT) sites^45^. To do that, the *cat* gene cassette containing FRT sites was amplified from the pKD3 plasmid using pKD3-Cat-pCDF-Forw and pKD3-Cat-pCDF-Rev primers and the pCDF-1B plasmid was amplified using pCDF 385-Rev and pCDF 425-Forw primers. The resulting PCR fragments were ligated together using the AMM kit so that the *cat* gene was inserted into the 385-425-base pair region of the pCDF-1B plasmid^43^. The Mat1A gene was then cloned into pCDF-Cat the same way as Mat1A was inserted in pCDF-1B vector and the resulting Mat1A-pCDF-Cat plasmid was used as template to amplify the Mat1A-FRT-cat-FRT fragment that was inserted into the *E. coli* genome (see below). The primers used for the cloning are listed in Table 2. All cloned genes were verified by sequencing.

### *E. coli* strains construction

*E. coli* strains K-12 MG1655, JW 1794-1 (Δ*fad::kan*) and JW2804-1 (Δ*aas::kan*) were used as the starting point for strain construction^46^. The SS19 strain carrying a double Δ*fad* Δ*aas* deletion was generated as previously described^11^. A Mat1A knock-in PCR fragment was generated by using the primers FadD KO – pCDF1 P1-1 and FadD KO – CAT P2-1 for amplification on the Mat1A-pCDF-Cat plasmid and further extended in a second round of PCR using the primers FadD-P1-pKD4-Primer2 and FadD-P2-pKD4-Primer2. This PCR fragment was employed to insert Mat1A into the fadD gene region of the K-12 MG1655 and JW2804-1 strains. Subsequent *cat* gene removal was performed according to protocol from Datsenko and Wanner^45^. A λDE3 prophage was integrated and BTE-pBAD/p15A and *Dm*JHAMT-pET15b plasmids were transformed into each strain. The list of strains and their genotypes are in Table 1.

### Protein Expression and Purification

DmJHAMT was expressed from *Dm*JHAMT-pET-28a(+) plasmid in a BL21(DE3) strain and purified using Ni-NTA affinity chromatography. 2 mL of an overnight starter culture was transferred to 2 L of LB media containing 50 μg/ml kanamycin and incubated at 37 °C. When the OD_600_ of the culture reached 0.6, isopropyl β-D-thiogalactoside (IPTG) was added to a final concentration of 0.5 mM and incubated at 18 °C with shaking for 24 h. The bacterial cells were harvested by centrifugation, resuspended in 100 mL of 50 mM Tris-HCl pH 7.5, 0.3 M KCl, 5% glycerol and 1 mg/ml lysozyme, incubated at 4 °C for 30 min with gentle shaking and stored at −80 °C. The frozen bacterial pellet was thawed, lysed by sonication and centrifuged (15,000 rpm, Sorvall SS34 Rotor 40 min, 4 °C). The lysate supernatant was incubated with 10 mL of Ni-NTA superflow resin at 4 °C for 30 minutes. The beads were washed 4 times with 10 mL of 50 mM Tris-HCl pH 7.5, 0.3 M KCl, 5% glycerol, 5 mM imidazole and the protein was eluted with 50 mM Tris-HCl pH 7.5, 0.3 M KCl, 5% glycerol, 250 mM imidazole. MTAN and LuxS were purified as described previously^47^,^48^. All proteins were dialyzed into 50 mM Tris-HCl pH 8.0, 20% glycerol, 0.2 M KCl solution and stored at −80 °C.

### Enzyme Assays

*Dm*JHAMT activity was measured using an enzyme-coupled colorimetric assay for SAM-dependent methyltransferases^49^. Enzyme assay solutions contained 20 μM LuxS, 10 μM MTAN, 500 μM SAM and various concentrations of fatty acid substrates in degassed 50 mM potassium phosphate [pH 8.0] at a final volume of 500 μL. 3 μM *Dm*JHAMT was used for the k_cat_/K_m_ calculations of lauric acid (Fig. 2b) and 10 μM *Dm*JHAMT was employed for reaction rate calculation with other fatty acids (Fig. 2a). Fatty acids were added from stock solutions prepared at 1 mg/mL in 100% ethanol. C16 palmitic acid was insoluble at concentrations >50 μM so comparison of the reaction rates for different fatty acid substrates was performed at 40 μM fatty acid. 5–200 μM range of lauric acid was used to obtain k_cat_/K_m_ values for this specific substrate^50^. All components of the assay except *Dm*JHAMT were combined and mixed and the reaction was initiated by addition of *Dm*JHAMT at 30 °C. 60 μL of the reaction mixture was taken out at various time points and quenched by adding 180 μL of 260 μM DTNB, 0.5 mM EDTA, 6 M GuHCl (room temperature) and the absorbance at 412 nm read after a 20 min incubation. A standard curve for SAH consumption by MTAN/LuxS was developed and used to quantify FAME production in the enzyme-coupled assays. All experiments were done in duplicate or triplicate and standard deviation from the mean value was used for error bars.

### SAM/SAH Assay

The SAM/SAH measurement protocol from cultures was modified from^51^. *E. coli* cells were pelleted by centrifugation (6000 rpm, Eppendorf F45-30-11 rotor, 5 min, 4 °C) and the wet cell weight was measured for each sample. The cells were resuspended and lysed by vortexing in 5% trifluoroacetic acid at 4 °C for 2 min (4 ml/g of wet cell weight). The cell lysate was clarified by centrifugation (13000 rpm, Eppendorf F45-30-11 rotor, 5 min, 4 °C) and 120 μl of supernatant was analyzed by high performance liquid chromatography (HPLC) as described in^51^. The concentrations were calculated using SAM and SAH standards of known concentrations. All measurements were performed in triplicate.

### Cell growth

Most of the strains did not reach saturation point in minimal media supplemented with either glycerol or glucose and terrific broth (TB) with 1.5% glycerol was used for cell growth and subsequent analysis. The media was supplemented with ampicillin (50 μg ml^−1^), chloramphenicol (34 μg ml^−1^) or kanamycin (50 μg ml^−1^) as appropriate. 5 mL of TB-glycerol were inoculated from a single colony and cultured overnight at 37 °C. The seed cultures were then used to inoculate 30 mL TB-glycerol medium with appropriate antibiotics in 150 mL culture tubes and cultivated at 25 °C in a rotary shaker (210 rpm). BTE and *Dm*JHAMT expression was induced at an OD_600_ of 0.1 with 50 μM Isopropyl-β-D-thio-galactoside and/or 0.002% L-arabinose. For samples with a dodecane overlay, 6 ml of dodecane were added after 24 hours of growth. Cultures were grown for an additional 1 day prior to FA/FAME analysis as described below.

### Metabolite extraction and identification

FFAs and FAMEs were extracted by addition of 6 mL of a 2:1 chloroform/methanol mixture (spiked with 0.15 mg/L of either methyl tridecanoate or methyl heptadecanoate as an internal control) to 5 ml of culture. For consistency in data analysis, 1 mL of dodecane layer was similarly treated with 6 mL of a 2:1 chloroform/methanol mixture before gas chromatography (GC) analysis. Quantification of FAs/FAMEs was conducted by GC-FID using an HP 5890 Series II gas chromatograph equipped with an HP-Innowax Column (0.32 mm x 30 m x 0.25 μm, Agilent). All samples were analyzed using the following parameters: inject: 1 μl; inlet temperature 250 °C with split ratio 1:1; carrier gas: helium; flow: 5 ml/min; oven temperature: initial temperature of 160 °C, hold 3 min; gradient to 255 °C at 5 °C/min; hold 3 min; inlet temp: 270 °C, detector temp: 330 °C. The amount of FAs/FAMEs was determined by comparison to a standard curve of various FAs and FAMEs and methyl tridecanoate or methyl heptadecanoate concentrations. To identify all FA/FAME products, GC/mass spectrometry analysis was additionally performed using an Agilent 6890-5975 equipped with HP-Innowax Column (0.32 mm x 30 m x 0.25 μm, Agilent). Peak identification was performed through comparison with GC retention time, known standards and mass spectra with the National Institute of Standards and Technology (NIST) database.

## Additional Information

**How to cite this article**: Sherkhanov, S. *et al*. Production of FAME biodiesel in *E. coli* by direct methylation with an insect enzyme. *Sci. Rep.* **6**, 24239; doi: 10.1038/srep24239 (2016).

## Figures and Tables

**Figure 1:**
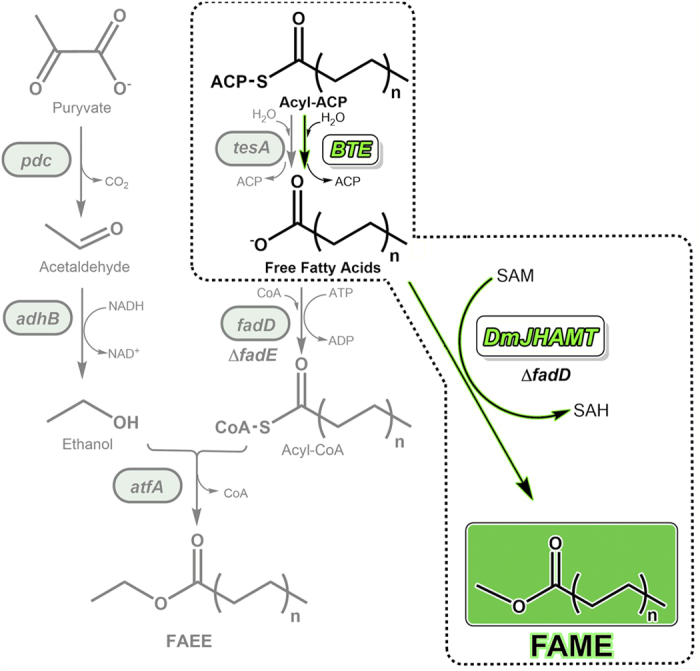
Two-step FAME pathway. The FAEE microdiesel pathway previously implemented in *E. coli* is outlined in the left panel in gray^3^,^7^. In the FAEE pathway, ethanol is produced by the introduction of pyruvate decarboxylase (*pdc*) and alcohol dehydrogenase (*adhB*) from *Zymomonas mobilus*. Acyl-Coenzyme A (CoA) thioesters are simultaneously produced by diverting fatty acid biosynthesis with the expression of various thioesterases (TES) and a yeast acyl-CoA ligase (ACL). Wax ester synthase (atfA) condenses the ethanol and acyl-CoA to make FAEE. In this study (dashed box, black/green), biodiesel is produced by the introduction of *Dm*JHAMT into a fatty acid producing strain. The medium-chain free fatty acid (FFA) pool is enriched in by expressing *U.californica* acyl-ACP thioesterase (BTE) in a β-oxidation-and phospholipid synthesis-deficient *E. coli* strain (Δ*fadD* Δ*aas*). Medium-chain FFAs are then methylated in *S-*adenosyl-*L-*methionine (SAM)-dependent manner to fatty acid methyl esters (FAMEs) by *Dm*JHAMT. Internal SAM levels are upregulated by introducing *S-*adenosylmethionine synthetase gene Mat1A from rat liver into *E. coli* genome. FA, fatty acid; ACP, acyl-carrier protein; GPE, glycerophosphoethanolamine; SAM, *S-*adenosyl-L-methionine; SAH, *S-*adenosylhomocysteine; FadD, acyl-CoA synthetase; Aas,2-Acyl-GPE acyltransferase/acyl-ACPsynthase; BTE, *U.californica* acyl-ACP thioesterase; Mat1A, rat *S-*adenosylmethionine synthetase; *Dm*JHAMT, *D. melanogaster* Juvenile hormone acid *O-*methyltransferase.

**Table 1:** *E. coli* strains used in this study.

Strain	Description/Genotype	Source
K-12 MG1655	F-, *λ,*^*−*^Δ*(araD-araB)567,* Δ*(rhaD-rhaB)568, hsdR514,* Δ*lacZ4787(::rrnB-3), rph-1,*	CGSC, New Haven, CT
JW 1794-1	K-12 MG1655 Δ*fadD730::kan*	CGSC, New Haven, CT
JW2804-1	K-12 MG1655 Δ*aas-761::kan*	CGSC, New Haven, CT
SS00	JW 1794-1 λ(DE3)	11
SS3B	SS00 p*Dm*JHAMT-pET15b, pUcBTE-pBAD/p15A (Δ*fadD* BTE/*Dm*JHAMT)	This study
SS4B	SS3B pMat1A-pCDF1B (Δ*fadD* BTE/*Dm*JHAMT/Mat1A)	This study
SS19	JW2804-1° Δ*fadD730::kan (*Δ*aas* Δ*fadD)* λ(DE3)	11
SS30	SS19 p*Dm*JHAMT-pET15b, pUcBTE-pBAD/p15A (Δ*aas* Δ*fadD* BTE/*Dm*JHAMT)	This study
SS33	SS3B *∆fadD*::Mat1A (*∆*fadD::Mat1A BTE/*Dm*JHAMT)	This study
SS34	SS30 Δ*fadD730*::Mat1A (Δaas ΔfadD::Mat1A BTE/*Dm*JHAMT)	This study

**Table 2:** Oligonucleotide Primers used in this study.

Name of Primer	Sequence
XhoI-pBAD/p15A-BTE	5′-GGGTTTTCTCGAGGAGTGGAAGCCGAAGCCGAA-3′
NciI-pBAD/p15A-BTE	5′ GGGTTTTATGCATTTACACCCTCGGTTCTGCGGGTA-3′
*Dm*JHAMT NdeI Forward	5′-GGGAATTCCATATGAATCAGGCCTCTCTATATCAGCAC-3′
*Dm*JHAMT XhoI End	5′-GGCCGCTCGAGTTAATTTATTCCCTTAACCAAGTTTTG-3′
5′ *E. coli* SAH NdeI	5′-GCATGGGAATTCCATATGAAAATCGGCATCATTGGTGCAATGG AAGAAGAAGTTAC-3′
3′ *E. coli* SAH SacI	5′-CAAGCTTGTCGACCGAGCTCTCATTAGCCATGTGCAAGTTTCTG CACCAGTGACTC-3′
5′ B. sub LuxS NheI	5′-CAAGCTTGATGGCTGCTAGCCCTTCAGTAGAAAGTTTTGAGCTT GATCATAATGCG-3′
3′ B. sub LuxS EagI	5′- GTGCGGCCGCGCCAAATACTTTTAGCAATTCTTCTTTATCCTGTG AAAAGCC-3′
5′ KpnI Mat1A forward	5′-GCCGGTACCATGAATGGACCTGTGGATGG-3′
3′ XhoI Mat1A end	5′-GCACTCGAGGCTTTACTAAAACACAAGCTTCTTGGG-3′
pKD3-Cat-pCDF-Forw	5′-GGCATTTGAG AAGCACACGG TCACAGTGTAGGCTGGAGCTGC TTC-3′
pKD3-Cat-pCDF-Rev	5′- CAGGGTCGTTAAATAGCCGCTTATG ATGGGAATTAGCCATGGT CC-3′
pCDF 385-Rev	5′-TGTGACCGTGTGCTTCTCAAATGCC-3′
pCDF 425-Forw	5′- CATAA GCGGCTATTT AACGACCCTG-3′
FadD KO – pCDF1 P1-1	5′- GACGACGAACACGCATTTTAGAGGTGAAGAAGGTTTTGCGCCA TTCGATGG-3′
FadD KO – CAT P2-1	5′- GATTAACCGGCGTCTGACGACTGACTTAACGCCAGGGTCGTTAA ATAGCCGC -3′
FadD-P1-pKD4-Primer2	5′- TATCATTTGGGGTTGCGATGACGACGAACACGCATTTTAG-3′
FadD-P2-pKD4-Primer2	5′-GCGTCAAAAAAA ACGCCGGATTAACCGGCGTCTGACGACTG-3′

The restriction sites are underlined.
